# Downregulation of angiogenic factors in aqueous humor associated with less intraoperative bleeding in PDR patients with NVG receiving conbercept: a randomized controlled trial

**DOI:** 10.1186/s12886-022-02451-6

**Published:** 2022-05-18

**Authors:** Qing Xu, Chaoju Gong, Lei Qiao, Ruifang Feng, Haiyang Liu, Yalu Liu, Liu Yang, Wei Fan, Lina Guan, Jie Li, Yipeng Zhang, Suyan Li

**Affiliations:** Department of Ophthalmology, Xuzhou Eye Disease Prevention and Treatment Institute, Xuzhou First People’s Hospital, The Affiliated Xuzhou Municipal Hospital of Xuzhou Medical University, 221116 Jiangsu Province, China

**Keywords:** Proliferative diabetic retinopathy, Neovascular glaucoma, Conbercept, Angiogenic factors, Intraoperative bleeding

## Abstract

**Background:**

To analyze the level changes of 28 cytokines in aqueous humor of patients with proliferative diabetic retinopathy (PDR) coexisting neovascular glaucoma (NVG) after intravitreal injection of conbercept (IVC), and to investigate whether these cytokines are associated with intraoperative bleeding (IOB).

**Methods:**

Totally 34 eyes with NVG secondary to PDR were enrolled. Patients were randomized into two groups, and all of them underwent 25-gauge pars plana vitrectomy (PPV) combined with trabeculectomy. Group I, 18 eyes received IVC 3 days before PPV, and 100 µL aqueous humor was collected at the time of IVC pretreatment and 3 days later at the beginning of PPV respectively. Group II, 16 eyes received IVC after PPV, and 100 µL aqueous humor was collected only at the beginning of PPV. Aqueous humor from 19 eyes with age-matched cataract patients served as controls. Luminex bead-based multiplex array was used to measure the levels of 28 cytokines in aqueous humor. The baseline cytokine levels were compared among the three groups. All NVG patients were divided into IOB and non-bleeding (INB) groups. The cytokine levels of aqueous humor at the beginning of PPV were compared between group I and II, also between IOB and INB groups. IOB in NVG patients was graded according to vitreous bleeding amount. The correlation between cytokine levels and the grades of IOB were analyzed.

**Results:**

Compared with controls, the baseline levels of 18 cytokines associated with inflammation and angiogenesis showed significantly increased in group I and group II (all, *P* < 0.0167). The IOB rate as well as the levels of IL-4, IL-22, Ang-2, PLGF and VEGF-A in group I were significantly lower than in group II (all, *P* < 0.05). The levels of IL-4, IL-22, Ang-2, PLGF and VEGF-A were significantly lower in INB group than in IOB group (all, *P* < 0.05). The levels of IL-4, Ang-2, PLGF and VEGF-A were positively correlated with the grades of IOB in NVG patients (all, *r*_*s*_ > 0.4, *P* < 0.05).

**Conclusions:**

IVC 3 days before PPV combined with trabeculectomy reduces IOB in NVG patients, in which the downregulation of IL-4, Ang-2, PLGF and VEGF-A after IVC may be an underlying mechanism.

**Trial registration:**

ChiCTR2100048118, retrospectively registered on 2 July 2021.

**Supplementary Information:**

The online version contains supplementary material available at 10.1186/s12886-022-02451-6.

## Background

Neovascular glaucoma (NVG) is a refractory glaucoma secondary to retinal ischemic disease [1]. Proliferative diabetic retinopathy (PDR) and central retinal vein occlusion (CRVO) are the most common causes of NVG [2]. Pathogenesis of NVG is the severe ischemia and hypoxia caused by retinal vascular disease, which induces the production and secretion of vascular endothelial growth factor (VEGF) by retinal pigment epithelial cells, Müller cells, astrocytes and so on. VEGF promotes the proliferation and migration of vascular endothelial cells, increasing vascular permeability and in turn boosting VEGF level in the vitreous and aqueous humor [3–5]. As a result, increased VEGF causes neovascularization at both iris surface and anterior chamber angle to compensate blood supply, leading to a close adhesion between peripheral iris and trabecular meshwork [6]. Thus, aqueous humor outflow is blocked to induce uncontrollable intraocular pressure (IOP) and intractable eye pain. Eventually, high IOP affects the blood supply for optic nerve and then causes irreversible visual damage [6].

At present, therapeutic strategy for NVG has been limited. The effects for simple filtration surgery and drainage valve implantation are often unsatisfactory. Intraoperative bleeding (IOB) and postoperative anterior chamber hemorrhage are important risk factors for surgical failure [7–9]. VEGF plays a key role in the occurrence of NVG [4]. Ohira et al. [10] and Kokubun et al. [11] both confirmed that patients with NVG had much higher VEGF levels in aqueous humor than those with other types of glaucoma. A number of clinical studies have shown that intravitreal injection of anti-VEGF agents can block the release of VEGF. As an auxiliary method in NVG therapy, anti-VEGF treatment provides a valuable time window for the successful implementation of anti-glaucoma surgery [12–14]. Anti-VEGF agents can promote the regression of intraocular neovascularization, reduce intraoperative and postoperative bleeding, and improve the success rate of surgery [3, 15].

Conbercept is a recombinant fusion protein that has a high affinity for all VEGF subtypes and placental growth factor (PLGF). It can competitively inhibit the binding of VEGF to its receptors and prevent activation of VEGF receptors [16]. Conbercept can treat PDR by inhibiting angiogenesis and inflammation [17–19]. At present, intravitreal injection of anti-VEGF agents is usually used in clinical practice to prevent IOB and postoperative anterior chamber hemorrhage during NVG operation. Our previous study showed that intravitreal injection of conbercept (IVC) 3 days before the operation significantly reduced the incidence of IOB and postoperative early anterior chamber hemorrhage in NVG patients compared with IVC after surgery [20]. However, the underlying mechanism has not been fully elucidated. Our study aimed to explore the mechanism of conbercept in reducing IOB during NVG operation and to provide a reference for the selection of NVG surgical plans.

## Methods

### Participants and grouping

**Fig. 1:**
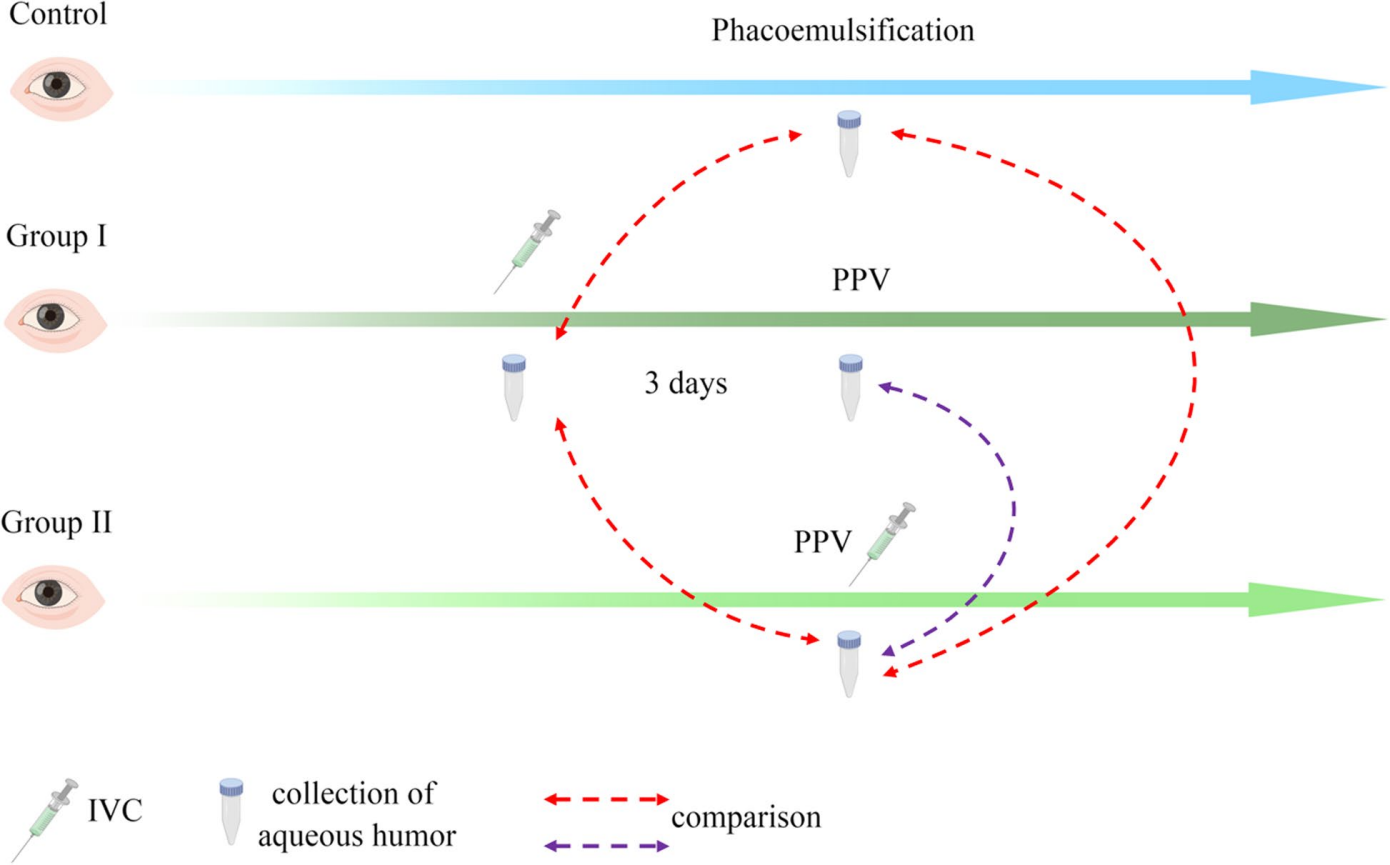
Study design and sample collection

This randomized controlled trial included 34 patients of 34 eyes diagnosed with stage III NVG complicated with vitreous hemorrhage (VH) in the Ophthalmology Department of Xuzhou First People's Hospital from February 2017 to August 2019. Patients were divided into two groups by random number table, and all of them underwent 25-gauge pars plana vitrectomy (PPV) combined with trabeculectomy. Group I, 18 patients of 18 eyes received IVC 3 days before PPV. Group II, 16 patients of 16 eyes received IVC after PPV. Nineteen patients of 19 eyes with cataracts who underwent phacoemulsification at the same time, age and gender matching, and without systemic diseases served as controls (Fig. 1). The inclusion criteria were as follows [3]: (1) NVG secondary to PDR; (2) never received intravitreal injection of anti-VEGF agents or panretinal photocoagulation (PRP) treatment; (3) IOP was higher than 21 mmHg but lower than 65 mmHg after using the maximum dose of an anti-glaucoma agent; (4) iris or angle neovascularization; (5) angle closure > 180°; and (6) age > 18 years. Exclusion criteria were as follows: (1) NVG secondary to other eye diseases; (2) active eye inflammation recently; (3) severe cardiovascular or cerebrovascular diseases making surgery intolerable; or (4) a history of drug allergy. This study was approved by the Ethics Committee of Xuzhou First People's Hospital (approval number: xzsdyyyll201610). All patients understood this study and voluntarily signed informed consent.

### Intravitreal injection of conbercept

All operations were performed by the same experienced physician in the operating theater. A 30G sterile disposable syringe needle was inserted into the eye through the pars plana (3.5–4 mm from the corneal limbus at baseline) to inject 0.5 mg/0.05 mL conbercept (KH902; Chengdu Kanghong Biotech Co., Ltd., Sichuan, China) into the vitreous.

### Twenty-five-gauge PPV combined with trabeculectomy

After routine sterilization and draping, the bulbar conjunctiva was incised with surgical scissors at the 5–7 o’clock position on the lower part of the eye. A scleral flap with the size of 4 mm × 5 mm and the thickness of 0.5 mm was prepared using a 3.0 mm scalpel. The scleral flap was applied with 25 mg/mL 5-fluorouracil cotton tablet for 5 min, and thoroughly rinsed with balanced salt solution. Three-channel 25-gauge PPV was performed. Phacoemulsification was performed when opacity of the lens obviously affected surgical field. During the operation, a 25-gauge vitrectomy cutter was used to excise the hemorrhagic vitreous. Triamcinolone acetonide (40 mg:1 ml; Kunming Jida pharmaceutical Co., Ltd., Yunnan, China) was locally injected to stain the posterior vitreous cortex. The preretinal fibrovascular membrane was excised, and panretinal photocoagulation (PRP) was performed. Gas or silicone oil was filled according to the degree of retinal detachment. 1 mm × 2 mm trabecular tissue was excised from the incision under the scleral flap, and peripheral iris was excised. The scleral flap and bulbar conjunctiva were fixed with 10–0 sutures. After the operation, cotton swab was used to massage the puncture site until no intraocular fluid leakage was found. Regular follow-up was performed. A fully automatic noncontact tonometer (TX-20; Canon Corporation of Japan) was used to measure the IOP at 3 day, 1 month, and 6 month after the operation. Best corrected visual acuity (BCVA) was recorded at 1 month and 6 month after the operation.

### Grading of IOB

Severity of IOB was defined as follows [21]: grade 0: no bleeding; grade 1: punctate bleeding with self-coagulation in a short time without hemostasis; grade 2: flaky bleeding may stop on its own, but interfere with the surgical procedures requiring aspiration with a flute needle; and grade 3: large amount of bleeding that required electrocoagulation hemostasis. According to whether IOB, NVG patients were divided into IOB group of 14 cases and non-bleeding (INB) group of 20 cases.

### Sample collection

Aqueous humor was collected at the time of IVC pretreatment and 3 days later at the beginning of PPV respectively in group I but only at the beginning of PPV in group II. Aqueous humor of cataract patients was collected at the beginning of phacoemulsification (Fig. 1). After topical anesthesia, 100 μL undiluted aqueous humor was withdrawn aseptically using an insulin syringe with a 30G needle at 1 mm inside the corneal limbus, which was placed in a 0.5 mL sterile Eppendorf tube and then stored at -80 °C until measurement.

### Measurement of cytokines

The levels of 28 cytokines associated with inflammation and angiogenesis were measured with Luminex bead-based multiplex array, including Eotaxin, interferon-γ (IFN-γ), interleukin 1b (IL-1b), IL-4, IL-5, IL-6, IL-8, IL-10, IL-12 (p70), IL-17A, IL-18, IL-22, IL-27, interferon-inducible protein-10 (IP-10), monocyte chemoattractant protein-1 (MCP-1), macrophage colony-stimulating factor (M-CSF), monokine induced by interferon-γ (MIG), platelet-derived growth factor AA (PDGF-AA), transforming growth factor α (TGF-α), tumor necrosis factor α (TNF-α), angiopoietin 2 (Ang-2), placental growth factor (PLGF), VEGF-A, epidermal growth factor (EGF), granulocyte colony-stimulating factor (G-CSF), Leptin, hepatocyte growth factor (HGF), and fibroblast growth factor 2 (FGF-2) (cytokine panels, HAGP1MAG-12 K and HCYTA-60 K-20C; Millipore Corporation, Billerica, MA, USA). All assays were performed strictly according to the manufacturer’s guidelines, the detailed procedures of which were described in our previous study [22]. The xPONENT FlexMAP 3D system on the Luminex platform was used to read the data. Milliplex analyst 5.1 software with a five parameter curve-fitting algorithm was used to analyze the data.

### Statistical analysis

Statistical analysis was performed with SPSS 19.0 (Statistical Product and Service Solutions, version 19.0, IBM). Shapiro–Wilk test was used to assess the normality of measurement variables. Normally distributed variables were expressed as mean ± standard deviation, whereas skewed distributed variables were expressed as median (Q1, Q3). Categorical variables were summarized as counts (percentages). One-way analysis of variance with post hoc least significant difference (LSD) multiple comparison tests was performed for normally distributed variables among controls and NVG patients. Comparisons of categorical variables were performed using chi-squared test or Fisher’s exact probability test. Decimal visual acuities were converted to LogMAR. The overall difference of BCVA and IOP between group I and II were assessed by two-way repeated measures analysis of variance with post hoc LSD multiple comparison tests. Kruskal–Wallis H test was performed for cytokine levels among controls and NVG patients of different groups. Mann–Whitney U test was performed for cytokine levels between two groups. To avoid the introduction of type 1 errors, the adjusted *P* value was divided by the number of comparisons (adjusted *P* = 0.05/3), and a calculated *P* < 0.0167 was considered significant for multiple comparisons. Spearman’s rank correlation test was performed to assess the association between cytokine levels and the grades of intraoperative bleeding. The correlation coefficient was tested by Student’s *t* test, and *P* < 0.05 was considered statistically significant.

## Results

### Demographic characteristics

There were no significant differences in age, sex composition, and eye composition among the three groups (all, *P* > 0.05, Table 1). The IOP was significantly higher in group I and group II than that of controls (*P* < 0.001), but there was no significant difference between two NVG groups (*P* > 0.05) (Table 1).

**Table 1:** Demographic characteristics of the study subjects and the control group

	Control (*n* = 19)	Group I (*n* = 18)	Group II (*n* = 16)	*P* value
Age	59.47 ± 12.27	55.56 ± 12.56	56.06 ± 9.44	0.541^a^
Men, n (%)	8 (42.11)	13 (72.22)	9 (56.25)	0.181^b^
Right Eye, n (%)	7 (36.84)	11 (61.11)	7 (43.75)	0.318^b^
Preoperative IOP (mmHg)	15.42 ± 3.37	46.06 ± 10.36^c^	45.88 ± 11.56^c^	0.000^a^

^a^ One-way analysis of variance with post hoc LSD multiple comparison tests were performed to compare the age and preoperative IOP among the three groups

^b^ The difference of the sex and eye distribution among the three groups were measured by χ^2^ test

^c^ Compared with control, *P* < 0.001

### Baseline cytokine levels

Compared with controls, The baseline levels of 18 cytokines showed significantly increased in group I and group II, including Eotaxin, IL-4, IL-6, IL-8, IL-18, IL-22, IL-27, IP-10, MCP-1, MIG, PDGF-AA, Ang-2, PLGF, VEGF-A, G-CSF, Leptin, HGF and FGF-2 (all, *P* < 0.0167). The levels of IFN-γ, IL-1b, IL-5, IL-10, IL-12 (p70), IL-17A, TGF-α, TNF-α and EGF were lower than the minimum detectable level of the panel. There was no significant difference in the levels of all the cytokines between group I and group II (all, *P* > 0.05) (Supplemental Fig. 1).

### Comparison of the clinical characteristics between groups I and II

The overall comparison of BCVA showed no significant difference between groups I and II at different time points (F_group_ = 0.086, *P* > 0.05; F_time_ = 0.716, *P* > 0.05) (Table 2). Even though there was no significant difference in the overall comparison of IOP between the two groups at same time point (F_group_ = 0.239, *P* > 0.05), the overall difference of IOP was statistically significant at different time points in each group (F_time_ = 151.895, *P* < 0.001). The postoperative IOP decreased at 3 day and increased at 1 and 6 month. The difference between any two time points was statistically significant (all, *P* < 0.05) (Table 3). In group I, 3 patients had IOB, 2 (66.67%) with grade 2, and 1 (33.33%) with grade 3. In group II, 11 patients had IOB, 2 (18.18%) with grade 1, 4 (36.36%) with grade 2, and 5 (45.45%) with grade 3. There was no significant difference in the IOB grades between the two groups (*P* > 0.05). The IOB rate in group I (3/18, 16.67%) was much lower than that in group II (11/16, 68.75%) (*P* < 0.05) (Table 4).

**Table 2:** Comparison of BCVA (LogMAR) between group I and II at different time points (means ± SD, number of letters)

Group	*N*	LogMAR BCVA at different time points		
		Baseline	1 month	6 month
I	18	2.01 ± 0.59	2.06 ± 0.57	2.14 ± 0.65
II	16	2.15 ± 0.48	2.07 ± 0.60	2.16 ± 0.70

Two-way ANOVA of repeated measurement with post hoc LSD multiple comparison tests was performed to compare the overall difference of BVCA between the two groups. F_group_ = 0.086, *P* = 0.771, F_time_ = 0.716, *P* = 0.442, F_interaction_ = 0.408, *P* = 0.586

**Table 3:** Comparison of IOP between group I and II at different time points (means ± SD, mmHg)

Group	*N*	IOP at different time points			
		Baseline	3 day	1 month	6 month
I	18	46.06 ± 10.36	11.06 ± 3.35^a^	17.56 ± 8.26^ab^	21.61 ± 9.24^abc^
II	16	45.88 ± 11.56	10.94 ± 2.49^a^	19.88 ± 9.04^ab^	23.25 ± 6.85^abc^

Two-way ANOVA of repeated measurement with post hoc LSD multiple comparison tests was performed to compare the overall difference of IOP between the two groups. F_group_ = 0.239, *P* = 0.628; F_time_ = 151.895, *P* <0.0001; F_interaction_ = 0.266, *P* = 0.850.

^a^ *P* < 0.05, compared with intragroup respective baseline value

^b^ *P* < 0.05, compared with intragroup respective postoperative 3 day

^c^ *P* < 0.05, compared with intragroup respective postoperative 1 month

**Table 4:** Comprasion of the incidence of IOB between group I and II [n (%)]

Group	*N*	IOB	
		Yes	No
I	18	3 (16.67)	15 (83.33)
II	16	11 (68.75)	5 (31.25)
*P*		0.004	

The difference of the IOB between group I and II was measured by Fisher's exact probability test

### Comparison of cytokine levels in the aqueous humor at the beginning of PPV between group I and II

**Fig. 2:**
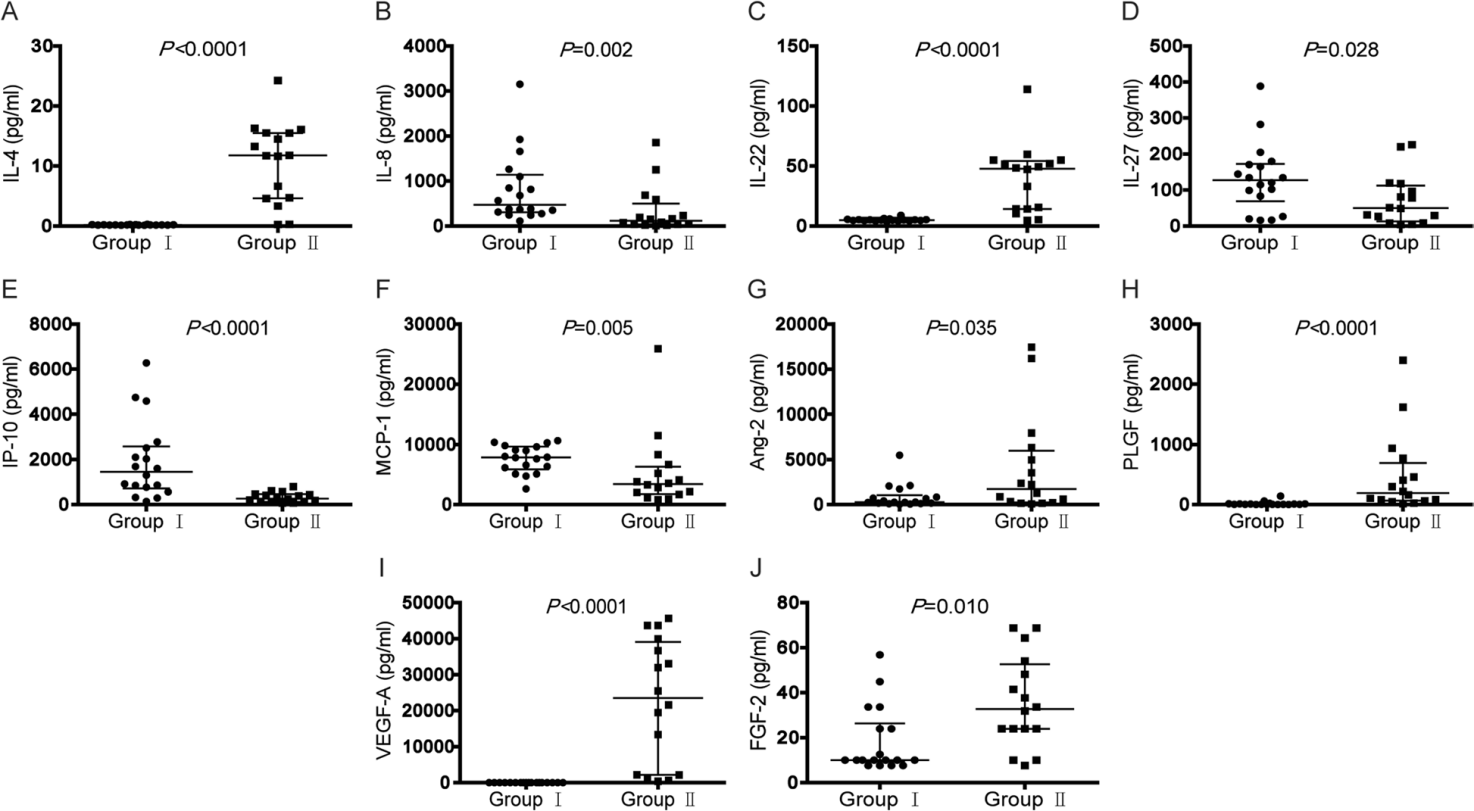
Comparison of cytokine levels in the aqueous humor at the beginning of PPV between group I and II A-J: The levels of IL-4, IL-8, IL-22, IL-27, IP-10, MCP-1, Ang-2, PLGF, VEGF-A and FGF-2 in the aqueous humor of group I and II were compared by Mann–Whitney test. *P* < 0.05 was considered statistically significant. Data were shown as median with interquartile range

The levels of IL-4, IL-22, Ang-2, PLGF, VEGF-A and FGF-2 were significantly lower in group I than in group II (all, *P* < 0.05), but the levels of IL-8, IL-27, IP-10 and MCP-1 were higher (all, *P* < 0.05) (Fig. 2). In addition, the levels of Eotaxin, IL-6, IL-18, M-CSF, MIG, PDGF-AA, G-CSF, Leptin, and HGF had no statistical significance between the two groups (all, *P* > 0.05) (Supplemental Fig. 2).

### Association between cytokine levels and IOB grades

**Fig. 3:**
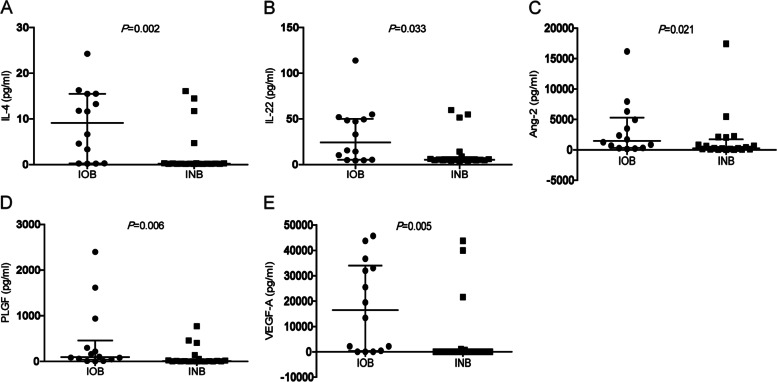
Comparison of cytokine levels in the aqueous humor at the beginning of PPV between IOB and INB group A-E: The levels of IL-4, IL-22, Ang-2, PLGF, VEGF-A in the aqueous humor of IOB and INB group were compared by Mann–Whitney test, *P* < 0.05 was considered statistically significant. Data were shown as median with interquartile range

The levels of IL-4, IL-22, Ang-2, PLGF and VEGF-A in the aqueous humor at the beginning of PPV were significantly lower in INB group than in IOB group (all, *P* < 0.05) (Fig. 3), the level of Eotaxin, IL-6, IL-8, IL-18, IL-27, IP-10, MCP-1, M-CSF, MIG, PDGF-AA, G-CSF, Leptin, HGF, and FGF-2 had no statistical significance (all, P > 0.05) (Supplemental Fig. 3). For all the NVG patients, correlation of IL-4, IL-22, Ang-2, PLGF, VEGF-A levels with the IOB grades were analyzed. The levels of IL-4, Ang-2, PLGF, VEGF-A but not IL-22 were positively correlated with the grades of IOB (all, *r*_*s*_ > 0.4, *P* < 0.05) (Table 5).

**Table 5:** Correlation of cytokine levels with IOB grades in NVG patients

Cytokines	Grades of IOB	
	*r*_*s*_	*P* value
IL-4	0.486	0.004
IL-22	0.326	0.060
Ang-2	0.409	0.016
PLGF	0.435	0.010
VEGF-A	0.489	0.003

Correlations between cytokine levels and the grades of IOB was estimated by Spearman’s ranked correlation

## Discussion

At present, the pathogenesis of NVG is still unclear. Its occurrence is mainly due to retinal vascular wall damage induced by ischemia and hypoxia, which promotes the production and release of VEGF into retina tissue and vitreous, consequently stimulating neovascularization [23]. Our study showed that VEGF-A level in aqueous humor was significantly higher in NVG than in controls, suggesting that VEGF-A plays an important role in the occurrence and development of NVG. In addition, the levels of other 17 cytokines, belonging to angiogenic factors, growth factors, and inflammatory factors, were also evidently elevated, which was consistent with previous findings [11, 24]. Here, the levels of IL-22, IL-27, MIG and Ang-2 were measured in NVG at the first time. These results suggest that various cytokines other than VEGF may participate in the pathogenesis of NVG by regulating neovascularization and immunoinflammatory response in the eye. Therefore, these cytokines may serve as new biomarkers and intervention targets for NVG.

Our study showed that the levels of angiogenic factors VEGF-A, PLGF, Ang-2 and anti-inflammatory factors IL-4, IL-22 were decreased after 3 days of IVC, while the levels of pro-inflammatory factors IL-8, IL-27, IP-10 and MCP-1 were increased. These results are consistent with previous findings that the levels of pro-inflammatory factors in PDR and NVG were increased after intravitreal injection of bevacizumab [25, 26], suggesting that conbercept not only reduces the levels of VEGF, but may also involve in other angiogenic and inflammation-related cytokine networks [27]. IVC did not change the levels of most growth factors, indicating the independent roles of different growth factors in NVG.

The direct effects of IVC on various cytokines in NVG remains unclear. The sustained high levels of growth factors and proinflammatory factors may be a compensatory responses to VEGF inhibition. Angiogenesis is mediated by a variety of cytokines, and the selective antagonism of one factor may result in a compensatory increase in other factors [10, 25]. The increase of pro-inflammatory factors and the decrease of anti-inflammatory factors after IVC may also result from the inflammatory response induced by the invasive intravitreal injection itself [10]. Thus, anti-VEGF therapy requires a safer and simpler route of administration to reduce tissue damage. In summary, a single IVC cannot completely reduce the levels of growth factors and pro-inflammatory factors in NVG aqueous humor, and the specific effects of IVC on these cytokines needs to be clarified in the future.

Intraoperative and postoperative bleeding are common clinical problems in retinal ischemic and hypoxic diseases such as PDR and NVG. IOB requires additional operations and increases the risk of retinal damage. Some cases of postoperative preretinal bleeding may also derive from the intraoperative bleeder that can't be cleaned up completely. Thus, IOB is the key factor influencing the prognosis of surgery [7, 8, 28]. Our study mainly focused on the effect of IVC before PPV on IOB in patients with NVG secondary to PDR. The results demonstrated that IVC 3 days before PPV significantly reduced the rate of IOB, which was consistent with previous studies that preoperative intravitreal injection of bevacizumab [28], ranibizumab [29, 30], or conbercept [21, 31] all reduced the IOB rate in patients with PDR, suggesting that different anti-VEGF agents have same function for reducing IOB in neovascularization diseases. However, earlier studies didn’t explain the potential mechanism of preoperative anti-VEGF to reduce IOB.

Our results indicated that the levels of angiogenic factors VEGF-A, PLGF, Ang-2 and anti-inflammatory factors IL-4, IL-22 decreased in NVG patients with IVC 3 days before PPV. Are these factors correlated with IOB? We grouped NVG patients according to IOB. Compared with IOB group, the levels of IL-4, IL-22, Ang-2, PLGF and VEGF-A were reduced in INB group. Further analysis showed that the levels of Ang-2, VEGF-A, PLGF, and IL-4 were positively correlated with the grades of IOB. As angiogenic factors, Ang-2, VEGF-A and PLGF play an essential role in PDR [32–34]. Peluzzo et al. summarized previous studies and showed that IL-4 is a pro-angiogenic interleukin albeit with ill-defined mechanisms [35], and Takashi et al. reported that IL-4 directly promoted choroidal neovascularization in age related macular degeneration mainly through IL-4R [36]. These evidences suggest that IL-4 may associates with neovascularization except for its anti-inflammatory role.

Our results indicated that, as an adjuvant treatment for NVG, IVC 3 days before PPV was necessary to reduce IOB and improve the success rate of surgery. Conbercept may reduce the incidence of IOB by down-regulating the levels of angiogenic factors Ang-2, VEGF-A, PLGF and IL-4. Our findings provide a theoretical basis for IVC before NVG surgery. However, considering the small sample size in this pilot study, multi-center, randomized, controlled clinical trial with larger sample size will be required to make our findings more solid.

In our study, the incidence of IOB reduced in NVG patients receiving IVC 3 days before PPV, which was consistent with previous studies. Yang et al. reported that IVC 3 days before the operation reduced the incidence and severity of IOB, and improved visual acuity at 3 day, 1 week, 1 month, and 3 month respectively in PDR patients [21]. Hu et al. found that the length and density of newly formed vessels in PDR were markedly reduced within 3 days after IVC, but at 5 or 7 days after IVC, the change is not significant [37]. However, due to that NVG is more serious than PDR, the timing for preoperative injection of anti-VEGF agents remains to be further investigated in NVG.

## Conclusions

Our study showed that IVC 3 days before 25-gauge PPV combined with trabeculectomy reduced the IOB in NVG, in which the downregulation of IL-4, Ang-2, PLGF and VEGF-A after IVC may be an underlying mechanism, confirming the necessity of preoperative anti-VEGF treatment for NVG surgery. Although conbercept is widely used in NVG, the definitive injection timing before NVG surgery and its solid theoretical basis has not been established. Our study explored the possible mechanism of IOB from the perspective of cytokines, which helps to optimize the preoperative treatment for NVG.

## Supplementary Information

**Additional file 1:** **Supplemental Figure 1.** Comparison of Baseline cytokines levels in the aqueous humor among 3 groups. **Supplemental Figure 2.** Comparison of cytokine levels in the aqueous humor at the beginning of PPV between group I and II. **Supplemental Figure 3.** Comparison of cytokine levels in the aqueous humor at the beginning of PPV between IOB and INB group.

## Data Availability

The datasets used and analyzed during the current study are available from the corresponding author on reasonable request.

## Abbreviations

PDR: Proliferative diabetic retinopathy
NVG: Neovascular glaucoma
IVC: Intravitreal injection of conbercept
IOB: Intraoperative bleeding
PPV: Pars plana vitrectomy
INB: Non- intraoperative bleeding
CRVO: Central retinal vein occlusion
VEGF: Vascular endothelial growth factor
IOP: Intraocular pressure
PLGF: Placental growth factor
VH: Vitreous hemorrhage
PRP: Panretinal photocoagulation
BCVA: Best corrected visual acuity
IFN-γ: Interferon-γ
IL-1b: Interleukin 1b
IP-10: Interferon-inducible protein-10
MCP-1: Monocyte chemoattractant protein-1
M-CSF: Macrophage colony-stimulating factor
MIG: Monokine induced by interferon-γ
PDGF-AA: Platelet-derived growth factor AA
TGF-α: Transforming growth factor α
TNF-α: Tumor necrosis factor α
Ang-2: Angiopoietin 2
EGF: Epidermal growth factor
G-CSF: Granulocyte colony-stimulating factor
HGF: Hepatocyte growth factor
FGF-2: Fibroblast growth factor 2
LSD: Least significant difference
