# Supplementary material for: Downregulation of angiogenic factors in aqueous humor associated with less intraoperative bleeding in PDR patients with NVG receiving conbercept: a randomized controlled trial
Source: BMC Ophthalmol. 2022 May 18;22:224. doi: 10.1186/s12886-022-02451-6 (PMC9115965; doi:10.1186/s12886-022-02451-6)
Supplement: Supplementary file 1 — Additional file 1: Supplemental Figure 1. Comparison of Baseline cytokines levels in the aqueous humor among 3 groups. Supplemental Figure 2. Comparison of cytokine levels in the aqueous humor at the beginning of PPV between group I and II. Supplemental Figure 3. Comparison of cytokine levels in the aqueous humor at the beginning of PPV between IOB and INB group. [file 12886_2022_2451_MOESM1_ESM.docx]

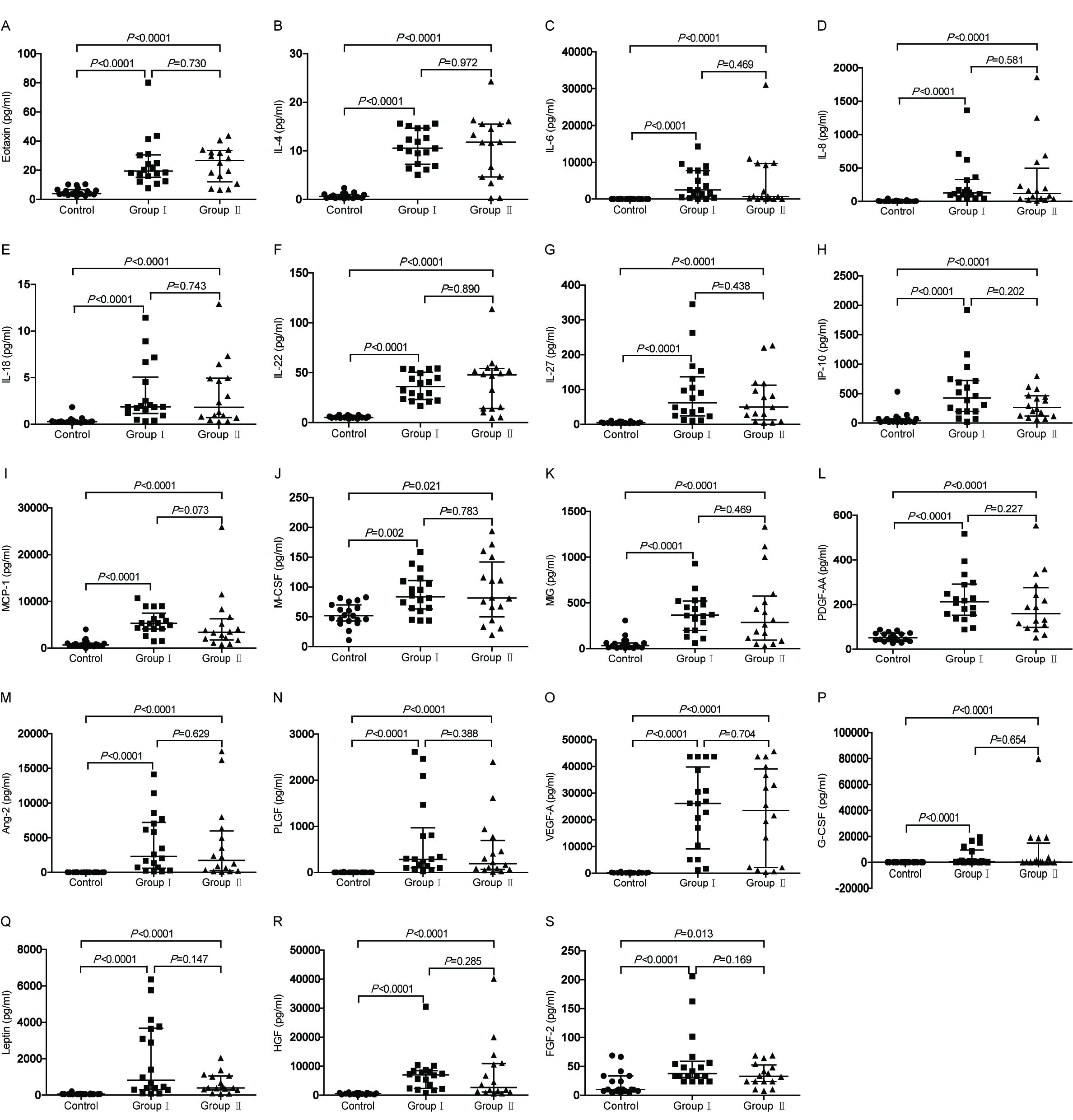


**Supplemental Figure 1 Comparison of Baseline cytokines levels in the aqueous humor among 3 groups**  A-S: The levels of Eotaxin, IL-4, IL-6, IL-8, IL-18, IL-22, IL-27, IP-10, MCP-1, M-CSF, MIG, PDGF-AA, Ang-2, PLGF, VEGF-A, G-CSF, Leptin, HGF, and FGF-2 in the aqueous humor of control, group Ⅰ, and Ⅱ were compared by Kruskal-Wallis H test; One-to-one multiple comparisons were performed by Mann-Whitney U test, *P*<0.0167 was considered statistically significant. Data were shown as median with interquartile range.


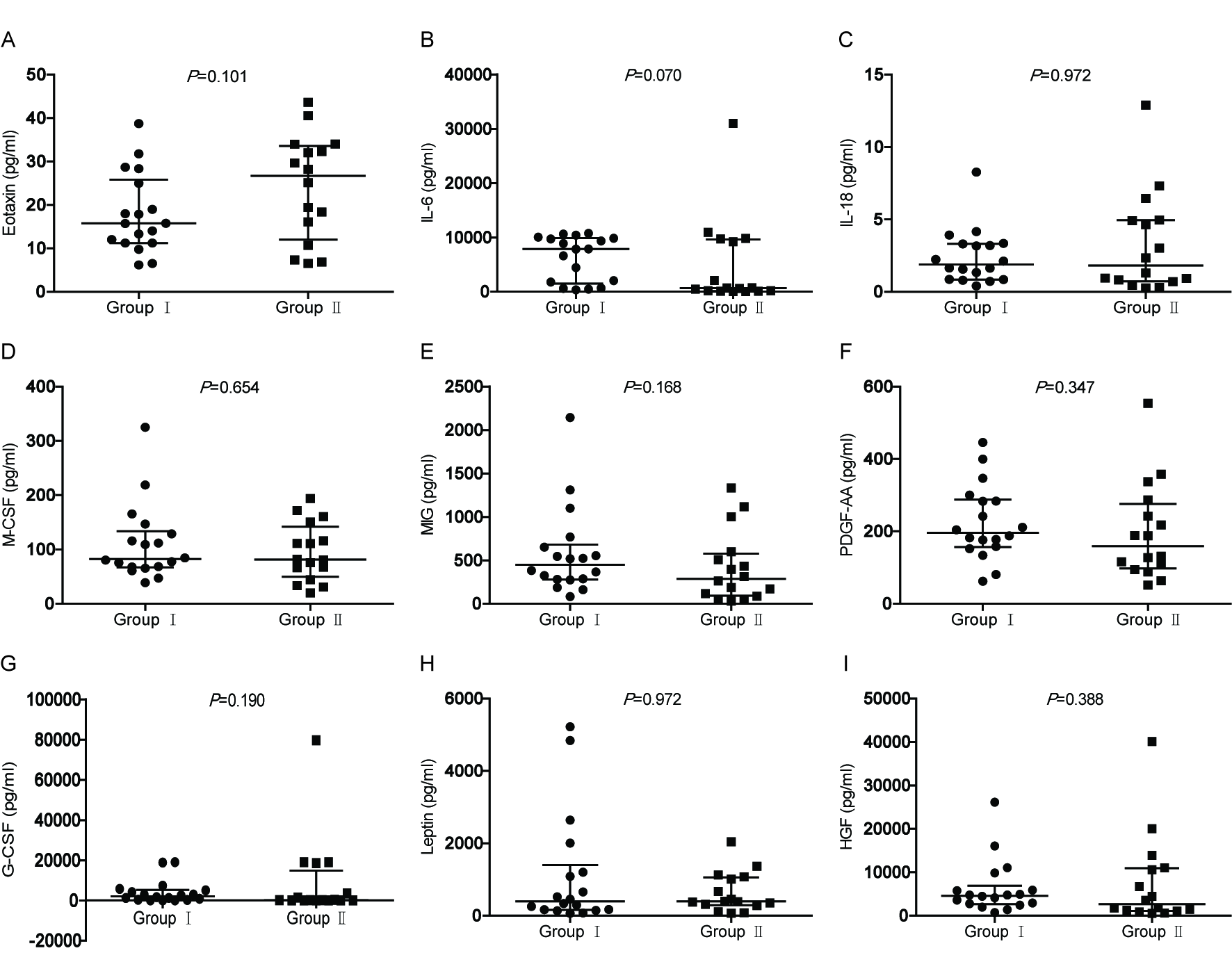


**Supplemental Figure 2 Comparison of cytokine levels in the aqueous humor at the beginning of PPV between group I and II** A-I: The levels of Eotaxin, IL-6, IL-18, M-CSF, MIG, PDGF-AA, G-CSF, Leptin and HGF in the aqueous humor of group Ⅰ and Ⅱ were compared by Mann-Whitney test, *P*<0.05 was considered statistically significant. Data were shown as median with interquartile range.


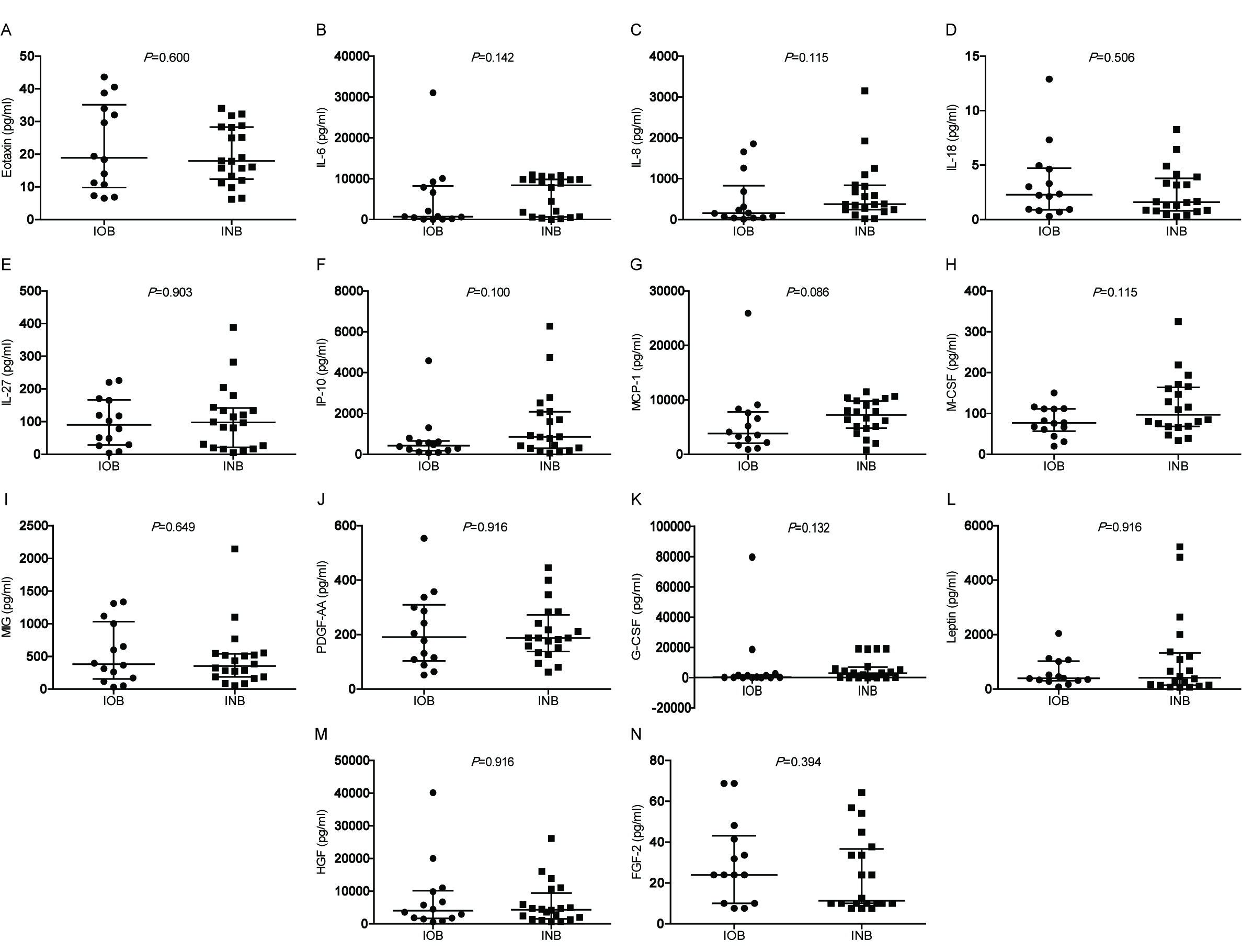


**Supplemental Figure 3 Comparison of cytokine levels in the aqueous humor at the beginning of PPV between IOB and INB group** A-N: The levels of Eotaxin, IL-6, IL-8, IL-18, IL-27, IP-10, MCP-1, M-CSF, MIG, PDGF-AA, G-CSF, Leptin, HGF, and FGF-2 in the aqueous humor of IOB and INB group were compared by Mann-Whitney test, *P*<0.05 was considered statistically significant. Data were shown as median with interquartile range.
